# Gynecological health and uptake of gynecological care after domestic or sexual violence: a qualitative study in an emergency shelter

**DOI:** 10.1186/s12905-024-03112-0

**Published:** 2024-04-27

**Authors:** Elisabeth Iraola, Jean-Pierre Menard, Isabelle Buresi, Patrick Chariot

**Affiliations:** 1 Institut de Recherche interdisciplinaire sur les Enjeux Sociaux (IRIS), UMR, CNRS U997 Inserm EHESS UFR SMBH, Université Sorbonne Paris Nord, 8156-997, 93300 Aubervilliers, France; 2 Direction de la protection maternelle et infantile et promotion de la santé, Conseil départemental du Val-de-Marne, 94000 Créteil, France

**Keywords:** Domestic violence, Intimate partner violence, Sexual violence, Pelvic examination, Gynecological care, Qualitative study

## Abstract

**Background:**

Domestic and sexual violence have been linked to adverse gynecological and obstetric outcomes. Survivors often find it difficult to verbalize such violence due to feelings of shame and guilt. Vulnerable or socially excluded women are frequently excluded from research, particularly qualitative studies on violence. This study aimed to characterize the perceived impact of domestic or sexual violence on the gynecological health and follow-up among women with complex social situations.

**Methods:**

We analyzed the data following inductive thematic analysis methods.

**Results:**

Between April 2022 and January 2023, we conducted 25 semi-structured interviews, lasting on average 90 min (range: 45–180), with women aged between 19 and 52, recruited in an emergency shelter in the Paris area. The women described physical and psychological violence mainly in the domestic sphere, their altered gynecological and mental health and their perception of gynecological care. The levels of uptake of gynecological care were related to the characteristics of the violence and their consequences. The description of gynecological examination was close to the description of coerced marital sexuality which was not considered to be sexual violence. Gynecological examination, likely to trigger embarrassment and discomfort, was always perceived to be necessary and justified, and consent was implied.

**Conclusion:**

This study can help question the appropriateness of professional practices related to the prevention of violence against women and gynecological examination practices. Any gynecological examination should be carried out within the framework of an equal relationship between caregiver and patient, for the general population and for women with a history of violence. It participates in preventing violence in the context of care, and more widely, in preventing violence against women.

**Supplementary Information:**

The online version contains supplementary material available at 10.1186/s12905-024-03112-0.

## Introduction

Since 2017, the international Metoo movement has contributed to freeing women’s voices about sexual violence [1–3]. However, domestic or sexual violence is still difficult to verbalize for survivors and associated with a lack of social and emotional support [4–7]. A controlled trial in the US showed that following sexual assault, survivors were more reduced to silence and stigmatized than after physical violence [8]. Several studies showed a link between a history of domestic or sexual violence and the occurrence of obstetric and gynecological symptoms or diseases that could encourage women to consult a health professional. Indeed, pain – pelvic pain, dysmenorrhea and dyspareunia – uterine bleeding, vaginismus, and endometriosis were increased among women reporting violence during childhood or adulthood compared to those who did not report such violence [9–13]. A majority of women survivors of violence who attend a consultation do not mention the violence to the caregiver, possibly because of the shame and guilt experienced after this violence [1, 14].

Several quantitative and qualitative studies showed the association between a history of domestic or sexual violence and a negative experience of pelvic examination [15–22]. Women with a history of domestic or sexual violence reported discomfort, embarrassment, shame, or an increased pain experienced during the gynecological examination compared to those who had not been subject to violence [15–22]. In 2022, we conducted a qualitative study that identified differences regarding the frequency of uptake of gynecological care for women surveyed by a feminist organization tackling sexual violence [23]. In that study, the differences in the uptake of gynecological care were associated to the characteristics of violence and its perceived effect on gynecological health. The participants in this study were all socially and professionally integrated. The study did not include any woman subject to complex social pathways [23]. Such women are often excluded from research, specifically qualitative studies about violence. The aim of this study was to characterize the perception of the impact of domestic or sexual violence on gynecological health and gynecological care among women likely to present with a vulnerable situation or facing social exclusion.

## Methods

The Consolidated Criteria for Reporting Qualitative Research (COREQ) guidelines were followed [24].

### Ethical considerations

The study was granted ethical approval from the ethical and research committee of Paris University (reference N°2021-82, October 12th, 2021). Informed consent was obtained from all individual participants included in the study and participants were assured of the confidential nature of the information collected.

### Study population

Semi-structured interviews were conducted with women recruited in an emergency shelter in the greater Paris area that welcomes women who have escaped violence, for a maximum of two months. Participants were approached using non-probability convenience and snowball sampling. Interviews were not conducted with women who presented acute anxiety, agitation, restlessness or confusion. When the research interview would alter psychic health by visibly reviving the trauma (flashbacks, acute anxiety), the interview was not initiated or continued. Interviews were not conducted with woman who had therapeutic expectations from the research interview, such as an exclusive need for psychological or gynecological care.

### Data collection

The individual face-to-face interviews lasted on average 90 minutes (range: 45–180) and were conducted by one of us (EI, midwife and PhD student), in French. Up to three pre-interviews were needed to establish a trusting relationship between the researcher and the participant. Interviews were audio-recorded and transcribed verbatim. At the beginning of the interview, some questions were asked so that the researcher could become familiar with the participants, gain their trust and create a safe atmosphere. Before the interview, the participants’ consent was obtained for participation and recording of the interview. Information was collected on their age, country of birth, level of education, and occupation. A semi-structured guide covering gynecological health, gynecological follow-up and domestic and sexual violence was used to shape interviews. Some examples of interview questions were as follows: “Could you tell me about your gynecological health?”, “What might lead you to consult a gynecologist or a midwife?”, “What is your perception of gynecological examination?” Could you tell me what brought you here?” (The interview guide is presented in Supplementary file 1).

Several listenings were needed to check the accuracy of the transcription. The transcribed data were compared with the field notes– e.g., non-verbal behavior was carefully transcribed during the interview– to ensure the completeness of the data collected. After verifying the accuracy of the transcription, recordings were destroyed. The data collection was ongoing until data saturation. The women interviewed were asked to identify themselves with a pseudonym.

### Data analysis

We analyzed the data following inductive thematic analysis methods, using NVIVO Version 10 [25, 26]. The recorded interviews were first carefully listened to and then transcribed verbatim. During the first stage, words, sentences or paragraphs of text were coded and combinations were formed using an inductive process. During the second phase, the final 5 interviews were used to confirm theoretical saturation across the data set, and to finalize the coding framework. During the third phase, we assembled the codes into potential themes and gathered all relevant data for each theme, checking the relevance of the themes in relation to the coded extract and the data-set as a whole. An example of a coded verbatim divided into themes and sub-themes is provided in Supplementary file 2. The reliability of the research was enhanced by the similarity of the results obtained individually, then cross-referenced by three authors (EI, JPM, PC), over the three stages, to limit subjectivity in data interpretation. The participants were involved in the research process to verify data consistency. Extracts of their data were shown in order to obtain their feedback, while taking care not to confront them with a verbatim report that was difficult to listen to or to read.

The professional backgrounds of the four authors, combining experience in sexual and reproductive health (EI, JPM, IB) and forensic medicine (PC), enriched data analysis, while ensuring that the sense of the participants’ words was not lost.

## Results

Between April 2022 and January 2023, 25 semi-structured interviews were conducted with women aged 19 to 52. Five women who had consented to participate in the study were lost to follow-up. The participants were in a vulnerable social situation or in a situation of social migration, had little or no academic qualification, or had a degree that was not acknowledged in France (Table 1). For some, speaking in French or English was difficult when it was not their mother-tongue. However, living in a shelter with other women helped them improve their French. Although an interpreter could have been called on if communication proved impossible, it was actually not needed. At the time of the interview, most of them declared having access to health insurance that ensured that their gynecological and obstetric care be state-funded.

**Table 1:** Degree, profession and length of residence in France

Nick Name	Degree	Occupation	Length of residence in France if born abroad	Age
Angelina	Bachelor’s in economy in the Comoros	Unemployed	7 years	30
Alicia	Associate degree in finance in Algeria	Unemployed	5 years	24
Ana	High school diploma in sales in France	Unemployed	Born in France	33
Jessica	No degree	Babysitter	4 years	38
Anaïs	No degree High school in France	Unemployed	Born in France	26
Karine	No degree	Unemployed	7 months	30
Louna	Professional training program in hotel management in France	Unemployed	Born in France	32
Christina	High school diploma in Algeria	Unemployed	13 years	53
Clara	Bachelor’s in sociology in Morocco	Unemployed	3 years	29
Cynthia	Accounting training in France	Unemployed	Born in France	27
Estelle	Laboratory employee training program in France	Unemployed	Born in France	33
Hélène	Bachelor’s in France	Air hostess	Born in France	52
Ingrid	No degree High school in France	Baby-sitter	Born in France	30
Iris	Bachelor’s in economy in Algeria	Unemployed	3 months	34
Irène	No degree	Unemployed	8 years	37
Justine	No degree	Unemployed	8 years	33
Katia	No degree	Unemployed	10 years	37
Lia	High school diploma in Tunisia	Unemployed	2 years	20
Lina	No degree	Unemployed	6 months	28
Lylia	Bachelor’s in French literature in Algeria	Unemployed	2 years	27
Marion	Personal assistant training program in France	Personal assistant	Born in France	29
Maya	Bachelor’s in communication in Congo	Waitress	3 years	32
Nora	Bachelor’s in management in Morocco	Unemployed	2 years	25
Stephania	Bachelor’s in human resources in Côte d’Ivoire	Unemployed	2 years	25
Sophia	Cooking class program in France	Unemployed	7 years	19

**Fig. 1:**
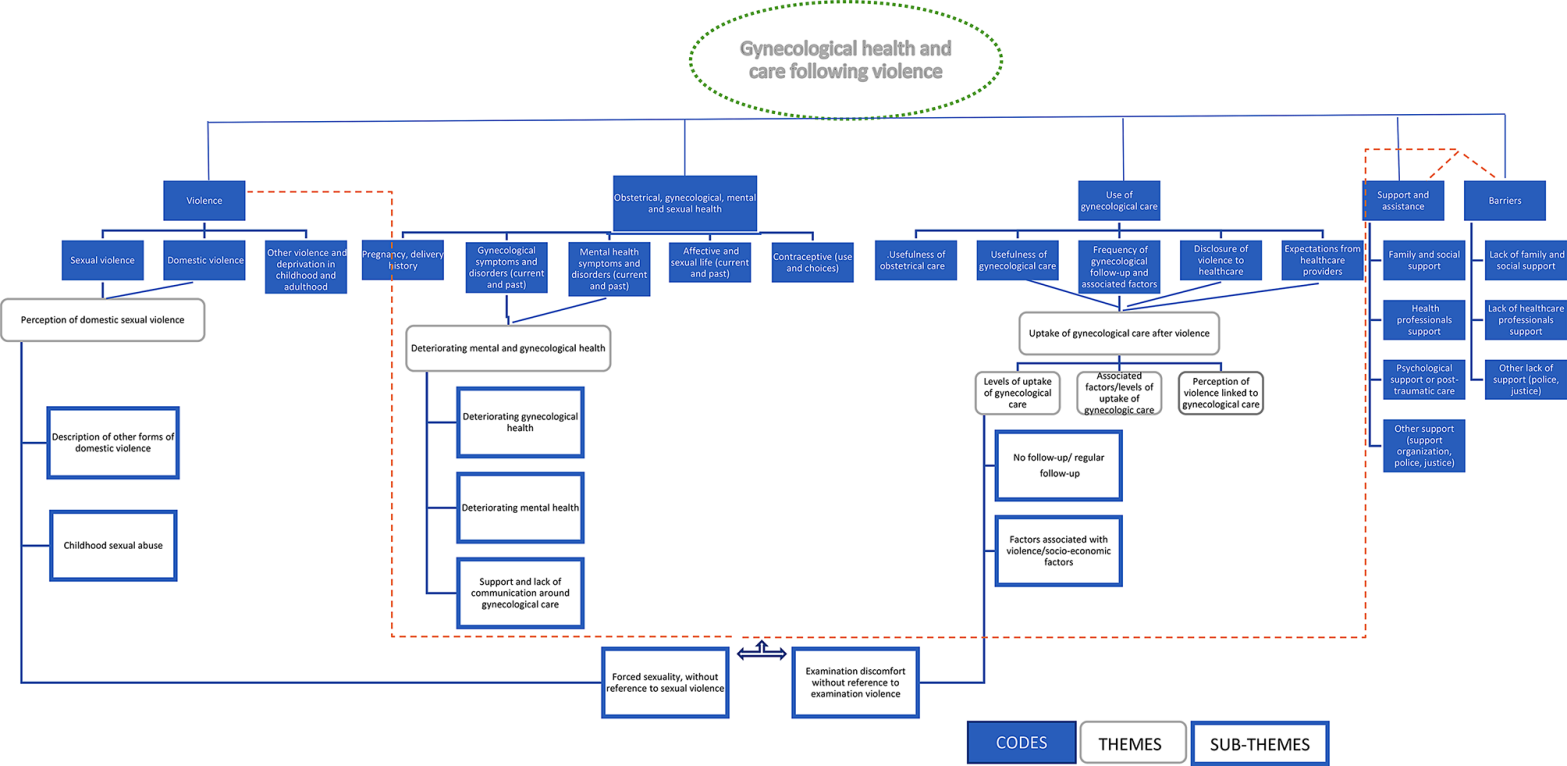
Thematic tree

The codes, divided into themes and sub-themes are presented in the thematic tree (Fig. 1).

### Theme 1: perception of domestic sexual violence

Women described a history of physical and psychological violence mainly in the domestic sphere. Violence was often associated to economic dependency, making leaving the home difficult. The last episode of violence was recent, and had taken place from a few days to a few weeks before the research interview. It was considered by the women to have been sufficiently serious to justify being housed urgently outside the marital home. They described the violence by closeantly referring to death.

I cannot forget that day. It was deadly (…) I was on the street. He was following me (*her husband*), he hit me. They came next (…) the police and the fire brigade. He insulted me and said *‘one day, I will slaughter you, there will be blood* (…) *with a knife’*. He said that sentence three days before hitting me. Every day I think of these words. Why does he talk that way? Every day he threatens me, every day, he insults me. And when he hit me, the neighbours called the fire brigade and the police. Me, I could not get up. I was shocked. He hit me with his hands and feet. Like that (*she mimes*). My face was swollen here and there (*she shows her face that still shows marks*). And then I can’t remember very well but I know the police and the fire brigade were there. The police said *‘Where does he live?* ‘. Even I was unable to speak. You see? Even while he hit me, I did not have the strength to push (…) I did not want him to touch my face. But it was in the face he was hitting me. My face was wrecked.

(Lia, 20 years old, unemployed)

In my mother’s flat, there is a small corridor. A very small corridor. And he hit me, he hit me (*her ex-husband*) (…) Like never before. In this tiny corridor, he did not stop (…) I could see in his eyes… I thought he was going to kill me (T*ears*) (…). In this corridor, there was a (…) how do you call it? A coat-rack (…) is that what you say? And with this and with everything else he would hit me. My daughter was there (…) She would yell ‘*Daddy, daddy, stop*’ (*Tears*) (…). He would kick me and punch me, in the face, breast and in the belly and on the back and then again on the belly. I heard a crack inside me. It was like (…) How do you say? As if my uterus cracked and tore. And then, bleeding, I bled a lot.

(Lylia, 27 years old, unemployed)

We argued and he grabbed my hair (…). We lived in a duplex, he threw me down the stairs. He hit me. To this day I still wonder ‘How *am I still alive? How am I still alive?*’. The neighbours heard. They heard every time but they did nothing, they were afraid of him. My neighbour told me *‘I heard, I thought of calling the police’*. I told her she should have. What if I had died?

(*Ana, 33 years old, unemployed)*

When questioned about sexual violence in the domestic sphere, they denied its existence. However, as they were asked to qualify their intimate and sexual life, they described it as forced or coerced, without referring to it as sexual violence. Following an event of physical domestic violence, sexual intercourse was perceived as what they referred to as ‘reconciliation’ and was considered more tolerable than physical violence, even when it was not desired.

He has needs. Sometimes he forced me but he never raped me.

(Irène, 37 years old, unemployed)

He forced me to do stuff, and it wasn’t just once. It was all the time (…). For me it was not rape. He was single, he was attracted to me and that’s it. He was a man, he had desires, and that’s it.

(Alicia, 24 years old, unemployed)

It’s the fact that he was rough, he hurt me. And in fact, he was bothered by my IUD, he said he could feel it. I felt like during penetration, he pushed it up or moved it. I said it was painful, I said I did not want to but my opinion did not count.

(Anaïs, 26 years old, unemployed)

I have come to a point where I have no sexual drive at all. I do it to avoid him insulting me. But I did not want to and I felt nothing (…) I cried, I did not want to do it (…) I felt nothing (…) When he touches me, I feel some (…) (*mimes disgust*). At least during the day, when he worked, I was relaxed. But at night, I could not escape it. It’s horrible (…) I do not want to have a sexual life anymore. At night I pretended to sleep. He comes inside me and I have to let him do what he wants. He does what he likes but I don’t want to (…). And I cry (…) And it disgusts me.

(Angelina, 30 years old, unemployed)

Afterwards, I could not block. But the body is hurt. During penetration, it was painful. You know, I could not stop him, it was not possible (…) And also (…) he touched me in front of the children. He touched my breasts and down there when the children were there, because at home, it was the only room, the children heard everything and saw everything (*Sobs*).

(Lina, 28 years old, unemployed)

I did not enjoy it. You see he could not penetrate there (…) So, he put his sex in my mouth. I said I did not like it but he kept going. And sometimes when he couldn’t put it in my mouth, he put in my bottom. I told him to stop but he did not stop. (*Tears*)

(*Nora, 25 years old, unemployed)*

While they did not name sexual violence in the domestic sphere, the participants described a chaotic and deprived childhood with violence, including sexual violence.

I was abused sexually when I was little. I am incapable of knowing when it started but in my first memories, I was 4 or 5. It was the son of my mother’s partner. Then, we were reported to child protection services but only for physical violence.

(Anaïs, 26 years old, unemployed)

There were repeated rapes by my step-father. I don’t know when it started but I must have been very little. Since I was little, I did not know that it was not normal, the rapes (…) I thought it was a game, so I said nothing. When I turned 10, I began asking myself questions *‘Is it normal? Is he allowed?*’ (…) No, it was not normal. I began to understand that it was bad. I started being aggressive, almost unbearable. I began my teenage crisis earlier than planned. Even my grandparents did not recognise me, as with them I was usually kind and peaceful (…). Then, I became aggressive even with them.

(Louna, 31 years old, unemployed)

#### Theme 2: deteriorating mental and gynecological health

The participants presented gynecological symptoms– pelvic pain, dyspareunia, dysmenorrhea, vulvodynia, bleeding, menstrual cycle disorders, infections– and psychological symptoms– anxiety, depression, eating disorders, addictions, ideation and suicidal behaviors, mental reminiscence. Pain (menstrual pain or dyspareunia) was a symptom that was often cited, associated to acknowledged sexual violence or to a sexuality described as unwanted, coerced or forced.

Gynecological symptoms

I regularly feel pain in my vagina. Intense pain, I don’t know, like it stings down there (…) and it can be really unbearable.

(Estelle, 33 years old, unemployed)

As M. (*her ex-husband*) had kicked me directly there (*she points to her pelvic area*) (…) I had bleeding before my period…and I had very very intense menstrual pain after that. I had my period last week, it was really unbearable. So so painful. Before, I had menstrual cramps, but normal. I could walk and do everything, not problem. There, I was like this (*she prostrates*) (…). You see?

(Iris, 34 years old, unemployed)

Psychological symptoms

I even lost (…) How to put it (…) lost all desire for anything. I lost the will to live (*Tears*). There are times where (…) I could see that I was rotten (…) I told myself that I could never be happy anyway, that I was the problem in my life. It was impossible to live like this, I was worthless.

(Angélina, 30 years old, unemployed)

(*Talking about her daughter*) She lives with her father in X (*Region*). I haven’t seen her for 5 years. She was 3 last time I saw her. My life has been very chaotic, too much so. Life caused me to abandon her as well as myself. I was very young when she was born, and I did not take the time to build myself before I became a mother. And then, when she was born, I was even more messed up than I am today. Today, I don’t think of death for example, whereas before I thought of it as the only way out. It was to disappear, and that is also why I disappeared from my daughter’s life.

(*Anais, 26 years old, unemployed)*

When I arrived here, I was so tired I could not walk. They immediately called ambulances and A&E. I had no strength, I ate nothing. Really nothing (…) I just smoked a lot (…) I did not sleep, I had nightmares. I wished I would die (*Tears*) (…) I wrote a letter… I wanted to die so that when they opened the letter, the legal services would reopen the case for domestic violence (*Sobs*).

(*Jessica, 38 years old, child minder*)

I was psychologically unwell, I did not sleep at night. Every time I heard some noises, I was startled. I also had fears, I felt he was coming in, threatening me, I still felt like I was with him. I still felt the effects (*or the facts*) in my body.

(Stephania, 25 years old, unemployed)

When they presented with gynecological symptoms, they complained about the lack of communication during the medical interview or the minimization of the gynecological symptoms by health professionals, which they associated with the violence.

I thought it’s weird, I have a problem, I feel nothing and it hurts. I am a robot, I feel nothing (…) nothing (…) nothing at all. I even consulted a gynecologist to explain it. I explained that I felt nothing during intercourse except for the pain. And he said *‘I don’t work on sexuality, you need to go and see a sex therapist*’. He asked when was the last time I took a smear test. He said it was to be done. But for sexuality, he (…) there was nothing that he could do.

(Angelina, 30 years old, unemployed)

Female genital mutilation was added to other violence and led to questions on the sexual dysfunctions imputable to one type of violence or the other– genital mutilation or sexual abuse – which they had never raised with a health professional.

When I was a little baby, I was mutilated. They removed my clitoris and everything was cut (…) closed. For birth, they opened up a little bit but never raised the issue. Because during intercourse, I feel no (…) never even (…). I never want to. It is very painful. Very often. I don’t know if it’s linked to this or to what was going on with my husband.

(Justine, 33 years old, unemployed)

Around the age of 8, they cut the… How is it called again? (…). And then, they sutured everything. I remember they were holding my legs. I remember the pain (…) I couldn’t even walk afterwards. To pee, it burnt, it was horrible. I stayed at home until it healed. Then after my first sexual intercourse, the first time, I think it tore. There was bleeding, pain and everything. (…). But I still have a question: even if your vagina is cut, do you remain a virgin?

(Sophia,19 years old, unemployed)

The integrity of the hymen, considered by women to be a proof of virginity, was raised when they reported the impact of the violence, as well as when they explained the motivation to access medical care, even a long time after the violence.

*I want to come back to what you said when we first met. You said ‘I am a virgin’. I feel like it is important to you to be so. Could you explain it to me?*

Yes, for us it’s the way it is, you cannot lose your virginity before marriage. And in fact, he (*perpetrator*] said that 50% of women don’t have a hymen. I think he said that (…) to (…) because with penetration and everything, I felt bad. So, he kept saying that the hymen, it didn’t exist, he said the hymen (…) Like it was bullshit (…) and I could have intercourse and remain a virgin.

(Alicia, 24 years old, unemployed)

(*Describing the gynecological examination*) When you have been subject to sexual violence, there can be lesions or problems. So, one should ask the question to find out and then check for any lesions. There can be internal vaginal tears. Damage in the genital area. Tears of the hymen (…) Small things like this. That’s what gynecological care is for. And sometimes, there can be multiple damages.

(Louna, 31 years old, unemployed)

#### Theme 3: uptake of gynecological care after violence

- Levels of uptake of gynecological care and associated factors.

The women surveyed showed a lack of knowledge and understanding of the care pathway in France, which led not to seek gynecological care. In addition, reduced or non-use of gynecological care was associated with avoidance of pelvic examination and coercive control by the perpetrator.

(*Avoidance*) And well, I don’t like when things are inserted in my vagina, like (…) cameras for example.

Cameras?

Yes, cameras to look. Or even fingers, or objects. I don’t want any object whatsoever.

(Alicia, 24 years old, unemployed)

(*Control*)

No (…) It was not possible (…) he was always behind me. Even during medical consultations, he is with me when I speak to a physician.

(Clara, 29, unemployed)

But my gynecologist, he was a man and my husband did not want me to go and see him (…) with him, it had to be a woman, not a man. Because a man, it was forbidden. Whereas for me, it does not matter because he is a physician and is doing his job. Man or woman, there is no difference. That’s also why I did not go back. It was too complicated.

(Ana, 33 years old, unemployed)

Limited financial resources associated with social vulnerability contributed to the decision not to seek gynecological care. In addition, factors such as low self-esteem and an inability to cope with the consequences of a diagnosis dissuaded women from seeking care. They did not wish to add negative events to an already chaotic and complex life.

Actually, I did not (…) uh (…) I left (…) actually, I had an appointment with a gynecologist but did not go (…) I let it go. (*Tears*). In fact, I did not care about myself, even if something happened, it was not a problem. I did not care for myself.

(*Ana*, *33 years old, unemployed*)

I think it was also because (…) well there are too many problems on my mind. It is not a good time. If there is a result and it is not good, I don’t have the strength to face it at the moment. I cannot have even one additional problem. At least I have nothing on that side. I have too much on my mind.

(*Jessica, 38 years old, child-minder*)

Regular uptake of gynecological care was easier during pregnancy. A dependency to the perpetrator for reproductive health could also lead women to seek gynecological care when they did not perceive the need.

It was my husband who wanted me to go (*for gynecological check-up*) because I couldn’t conceive any children (…) (*Speaking of her husband*) He said that at his age, it was not normal to only have 3 children. That we needed to have more v I did not want any more children. He was not himself. He changed completely. He said words that still hurt. Slaps too, more and more often. He wanted to show that he was the man and made the decision.

(Justine, 33 years old, unemployed)

b. Perception of violence linked to gynecological care.

The description of a gynecological examination was close to that of coerced marital sexuality which was not considered to be sexual violence. The gynecological examination, likely to trigger discomfort was always perceived to be necessary and justified, and consent was implied. When the physicians considered the gynecological examination necessary, their consent or approbation was not required.

He asked to get undressed and every time, even though I know the answer, I ask *‘Do we take only the top off or everything? Should I remove my underwear too?*’ (*Laughter*). I do it even though I know that everything must come off (…) but well when the physician goes (…) he puts his fingers or something else (…) legs spread you let go (…). And then, I close my eyes to escape a little (…). I close my eyes and let go (…) (*Laughter*).

(Cynthia, 27 years old, unemployed)

Am I asked permission? (…) Well then (…) that is a good question (*Laughter*) (…). In truth, that is what you go there for, isn’t it? But if you refuse, we say *‘no*’ anyway? On the other hand, they will not force you to undress either (…). For me, a physician is a physician, man or woman by the way. I think that you change as you grow old too. I believe that well they chose that job, and they do it for medical reasons (…) but well, a pair of breasts is a pair of breasts.

(*Hélène, 50 years old, air hostess*)

It was the first time I had an examination, he used a tube like for ultrasounds, and he put it in my vagina. It’s a bit like penetration honestly (*Laughter*). I had never seen this, it was weird. I thought that an ultrasound was just on the belly I did think that there was also a think on the other side (*Laughter*).

(Stéphania, 25 years old, unemployed)

## Discussion

The women reported physical and psychological violence mainly in the domestic sphere. They also described an altered gynecological and mental health and their perception of gynecological care. The levels of uptake of gynecological care were related to the characteristics of the violence and their consequences. When questioned about domestic sexual violence, they denied its existence. However, as they were asked to qualify their intimate and sexual life, they described it as forced or coerced, without referring to it as sexual violence. The description of gynecological examination was close to the description of coerced marital sexuality, which was not considered to be sexual violence. Gynecological examination, likely to trigger embarrassment and discomfort, was always perceived to be necessary and justified, and consent was implied. Their husbands’ sexual needs or the medical practitioner’s needs to perform a gynecological examination outweighed their own needs and therefore did not require their approval or consent.

While the perception of the impact of violence on gynecological health is comparable between women surveyed by a feminist organization (FO) [23] and those recruited in an mergency shelter (ES), there was a difference in the perception of domestic sexual violence and perception of violence during gynecological care. Thus, FO women reported domestic sexual violence while ES women did not consider forced sexuality in the context of physical and psychological violence as sexual violence. Their sexuality aimed at addressing their partner’s needs rather than their own emotional and sexual needs. The FO women also reported sexual violence in gynecological care [23], as previously shown by a Swedish study establishing that women with a history of violence during childhood were more likely to perceive care as violent [27]. Conversely, ES women described unease and discomfort, without referring to violence in gynecological care, which was described as useful and justified, which means that consent was implied.

We can assume that the perception of domestic sexual violence and violence in gynecological care is likely to vary depending on the timing of the violence, its repeated nature, and emotional, social and psychological support. This perception can also vary depending on education (egalitarian or stereotyped) and on a sense of belonging to a feminist culture as opposed to exclusive references to a patriarchal model.

Taking gynecological care or not can result from a choice, which contrasts with the impossibility of expressing free choices when life is controlled by domestic and sexual violence.

Data regarding mental health in our study corroborate the findings of several studies showing the link between domestic or sexual violence and disorders or symptoms such as depression, particularly postnatal depression [28–32], posttraumatic stress disorder [29–31], addictive behaviors [33], eating disorders [31, 34] and suicidality [31, 35]. The women interviewed in the present study regretted a lack of communication during the medical interview or a minimization, by health professionals, of the gynecological symptoms attributed to the violence, similarly to the women recruited by an FO [23]. Our results on the levels of gynecological care strengthen the findings of qualitative studies identifying the differences in levels of uptake of gynecological care in connection with the characteristics of the violence and their perceived effect on gynecological health [23, 36, 37].

Our study can help question the appropriateness of professional practices related to the prevention of violence against women. In France, the National Health Authority recommended screening for domestic violence [38], as did international learned societies [39, 40]. However, there is no French medical recommendation on screening for a history of sexual violence in adult women, as opposed to other countries like the US [41, 42]. This study also led to question the appropriateness of practices around gynecological examination. In the US, the UK and Australia, the indications of pelvic examinations are well defined [43–45]. Any gynecological examination should be carried out within the framework of an equal relationship between caregiver and patient, as well among women with a history of violence as in the general population.

## Strengths and weaknesses

Recruitment via organizations enabled to interview people in a neutral research setting rather than in the context of care and led to integrating women with difficult seek to gynecological care. Participants talked about their perception of the impact of violence on their health and gynecological care, topics which are usually ignored in care contexts, due to their association with guilt and shame. The study helped interview women with complex social pathways, usually excluded from any research on violence. We explored public health concerns regarding gynecological follow-up after domestic and sexual violence, including the interactions between biological, psychological, social and environmental factors.

Therefore, we cannot generalise the validity of the conclusions of this study to the general adult female population living in France. Future research is needed to understand, on the one hand, the uptake of gynecological care violence and, on the other hand, the social and psychological factors associated with the experience of domestic sexual violence and gynecological violence.

## Conclusions

Patients’ adherence to a medical act, such as a pelvic examination, is a critical element of the healthcare relationship, based on a clear understanding of the reasons for the examination and the conditions under which it will be performed. It contributes to avoid retraumatization, participates in preventing violence in the context of care, and more widely, in preventing violence against women.

### Electronic supplementary material

Below is the link to the electronic supplementary material.

Supplementary Material 1

Supplementary Material 2

## Data Availability

The datasets used and/or analysed in relation to the current study are available from the corresponding author upon reasonable request.

## Abbreviations

A&E: Accident and Emergency
ES: Emergency shelter
FO: Feminist organization
IUD: Intrauterine device
