# Supplementary material for: Gynecological health and uptake of gynecological care after domestic or sexual violence: a qualitative study in an emergency shelter
Source: BMC Womens Health. 2024 Apr 27;24:264. doi: 10.1186/s12905-024-03112-0 (PMC11055245; doi:10.1186/s12905-024-03112-0)
Supplement: Supplementary file 1 — Supplementary Material 1 [file 12905_2024_3112_MOESM1_ESM.docx]

**Interviews guide**

Introduce the study and create a climate of trust. The questions in the interviews guide are indicative only, and will be adapted both in wording and chronology to the women's stories. The interviews will be conducted with kindness, tact and caution, as they concern intimate and sensitive subjects.

1. Introduction
2. Presentation
3. Characteristics and perception of gynecological/obstetrical history and use of health care services

Sample questions (*for women who have already been pregnant*) *Could you tell me about your pregnancy(s) (pregnancy, delivery, abortion, miscarriage)?*

1. Gynecological, mental health and contraceptive preferences

Sample questions *Could you tell me about your gynecological health?*

*How do you feel since you've been here?* *And before living here. How did you feel?*

*Could you tell me about your previous/current method(s) of contraception? Have you ever used any method of contraception?*

1. Characteristics of violence, screening for violence and support

Sample questions *Could you tell me how you came to be here (in an emergency shelter), Could you tell me about the violence you have suffered?*

*Did you receive any help or support following the violence?*

1. Perceptions and expectations of gynecological care

Sample questions *Have you consulted for gynecological needs outside of pregnancy? Could you tell me about it?* *The gynecological examination. What can you tell me about it?*

**Additional file 1:** interview guide
