# Supplementary material for: Gynecological health and uptake of gynecological care after domestic or sexual violence: a qualitative study in an emergency shelter
Source: BMC Womens Health. 2024 Apr 27;24:264. doi: 10.1186/s12905-024-03112-0 (PMC11055245; doi:10.1186/s12905-024-03112-0)
Supplement: Supplementary file 2 — Supplementary Material 2 [file 12905_2024_3112_MOESM2_ESM.docx]

| Meaning unit | | | Codes | | Theme | Sub-themes |
| --- | --- | --- | --- | --- | --- | --- |
| I thought it’s weird, I have a problem, I feel nothing and it hurts. I am a robot, I feel nothing (…) nothing (…) nothing at all. I even consulted a gynecologist to explain it. I explained that I felt nothing during intercourse except for the pain. And he said ‘I don’t work on sexuality, you need to go and see a sex therapist'. He asked when was the last time I took a smear test. He said it was to be done. But for sexuality, he (…) there was nothing that he could do. | | | Affective and sexual life (current and past) | | Associated factors/levels of uptake of gynaecological care | No follow-up/regular follow-up |
|  |  |  | Gynecological symptoms and disorders (current and past) | |  | Factors associated with violence/socio-economic factors |
|  |  |  | Expectations from healthcare providers | |  |  |
|  |  |  |  | | | |
|  |  |  | |  |  |  |

**Additional file 2**: Example of data analysis
